# Quality-by-Design Principles Applied to the Establishment of a Pharmaceutical Quality Control Laboratory in a Resource-Limited Setting: The Lab Water

**DOI:** 10.1155/2022/2062406

**Published:** 2022-04-21

**Authors:** Sultan Suleman, Sileshi Belew, Dereje Kebebe, Markos Duguma, Henok Teshome, Gemmechu Hasen, Evelien Wynendaele, Luc Duchateau, Bart De Spiegeleer

**Affiliations:** ^1^Jimma University Laboratory of Drug Quality (JuLaDQ) and School of Pharmacy, Jimma University, PO Box 378, Jimma, Ethiopia; ^2^Drug Quality and Registration (DruQuaR) Group, Faculty of Pharmaceutical Sciences, Ghent University, Ottergemsesteenweg 460, Ghent B-9000, Belgium; ^3^Biometrics Research Group, Faculty of Veterinary Medicine, Ghent University, Salisburylaan 133, Merelbeke B-9820, Belgium

## Abstract

Quality-by-design (QbD) is defined as a systematic approach to design and develop a product/service based on sound science and quality risk management. It is already frequently applied in the pharmaceutical industry mainly in the development of pharmaceutical products and analytical methods but is not well established in the setup of facilities like quality control (QC) laboratory (lab). Therefore, lab QbD (lQbD) concept is introduced considering lab water purification system as an example. The water purification system comprising distillation unit coupled with Nanopure Analytical Ultrapure Water System combined with a 0.2-micron filter was established in Jimma University Laboratory of Drug Quality (JuLaDQ). The consistent capability of the established water purification system was evaluated through routine monitoring of the critical quality parameters (i.e., physicochemical, HPLC-DAD chromatogram total peak area, and resistivity) of freshly prepared lab water for a period of one year. In addition, quality of different grade water (tap water, distilled water (before and/or after cleaning distillation unit), and fresh ultrapure water (18.2 MΩ × cm at 25°C)) used in JuLaDQ was evaluated. The results of routine analysis of water quality revealed that HPLC global peak area at 210 and 254 nm could serve as one of the discriminatory control strategies to evaluate the capability of water purification system to produce the desired quality of lab water; and thus, we proposed a specification limit of 5,000 mAU∗s and 5,500 mAU∗s for global peak area at 254 and 210 nm, respectively, as system suitability parameter.

## 1. Introduction

The term quality-by-design (QbD) was created in 1970s by the quality expert Joseph *M* Juran and popularized in the 1990s [1]. Within the pharmaceutical field, International Conference on Harmonization (ICH) Q8(R2) defines QbD as a systematic approach to development that begins with predefined objectives and emphasizes product and process understanding and process control based on sound science and quality risk management [2]. United States (US) Food and Drug Administration (FDA) describes QbD as one arm of the quality system based on building quality in the development phase and throughout a product's life cycle [3].

The principles for the successful implementation of QbD for product development involve identification of the product attributes, such as a quality target product profile (QTPP) and critical quality attributes (CQAs); design space (the relationship between process inputs and CQAs); a robust control strategy to ensure consistent process performance; and finally; ongoing monitoring to ensure robust process performance over the life cycle of the product [4, 5]. ICH Q8 (R2) defines QTPP as a prospective summary of the quality characteristics of a drug product that ideally will be achieved to ensure the desired quality, taking into account safety and efficacy of the drug product. QTPP forms the basis of design for product development. Once QTPP has been identified, the next step is to identify the relevant CQAs. A CQA is defined as a physical, chemical, biological, or microbiological property or characteristic that should be within an appropriate limit, range, or distribution to ensure the desired product quality [2].

The QbD principle applied to product development is called product QbD (pQbD). These same QbD principles have also been applied to the development of analytical methods [6–9]. The concept of QbD applied to analytical method development is known as analytical QbD (aQbD) [10]. Equivalent to pQbD, aQbD plays a key role in the pharmaceutical industry for ensuring the product quality. Analytical QbD has different tools such as analytical target profile (ATP) establishment, CQAs, risk assessment, method optimization, and development with design of experiment (DoE), method operable design region (MODR), and control strategy [11]. It helps in the development of a robust and fit-for-purpose analytical method [12].

The ATP and MODR parallel the QTPP and design space defined for a product and its manufacturing process. MODR is a multidimensional space based on the method factors and settings that provide suitable method performance [6, 12–15]. DoE incorporates a set of characteristics which are essential in aQbD and is used for screening of factors, process characterization, and optimization of multiple responses [16, 17]. Therefore, the steps, tools, and approaches developed for application of QbD to manufacturing processes have analogous application in the analytical environment [18].

Applying the principles and concepts of pQbD and aQbD, a risk-based and robust quality management system can be built into quality control (QC) laboratories (labs) starting from establishment to provide enhanced flexibility and continuous improvement [15, 19] by reducing variations and producing consistent results. QC labs should generate reliable and traceable analytical quality data that meet user requirement specifications (URS). To ensure this, the lab needs a well-founded, effective, comprehensive, and defensible quality system in place [20, 21]. To establish such a system, prior knowledge of attributes that critically affect quality of analytical results of the QC lab is important. Literature indicates that human factors, accommodation and environmental conditions, methods, equipment, sampling and sample preparations, and handling of analytical procedures are some of the critical attributes [20, 22, 23]. Understanding these attributes and organizing them into a quality system can benefit a scientific risk-based approach. Even though information is scarce with regard to the application of such risk-based QbD approaches in pharmaceutical QC labs, there are indications of the usefulness of risk-based approaches to define analytical quality in clinical lab medicine [24, 25]. Therefore, this study was aimed to introduce lab QbD (lQbD) concept applied in the establishment of JuLaDQ considering lab water purification system as an example and was developed in the framework of a PhD thesis [26].

## 2. Methods

### 2.1. Establishment of a QC Lab

When the issue of establishing JuLaDQ came into picture, central strategic questions were first defined. (1) What is the purpose of the QC lab? (2) What standards are required? (3) What are the lab user requirements? Based on this, the required regulatory standards [27–29], the purpose of the QC lab (provision of QC-analytical services), the existing setup and risks associated with critical lab quality attributes were considered. The lab quality attributes that could have a risk in the performance of JuLaDQ were identified and used to design the QC lab workflow [30], based upon which JuLaDQ was physically established and became a running pharmaceutical QC lab. Analogous to pQbD and aQbD, lQbD was thus defined and formally recognized during prequalification inspection by the WHO inspection team [31].

### 2.2. Water Purification System in JuLaDQ

Since a single water purification unit operation process could not consistently and with sufficient robustness provide water *R* quality requirements [32], which is a minimum lab water quality target in JuLaDQ, a customized water purification system combining feasible, cost effective, and setting-suitable purification technologies was established. The established water purification system comprises distillation unit (Water Still Bibby W4000, UK) coupled with Nanopure Analytical Ultrapure Water System (model number: D11901 (7143), Thermo Fisher Scientific) combined with a 0.2 micron filter (Barnstead D3750).

### 2.3. Experimental

#### 2.3.1. Materials and Reagents

Distilled water and ultrapure water (18.2 MΩ × cm at 25°C) produced in JuLaDQ and tap water (running water) were used. The following were used: acetonitrile (HPLC grade, Sigma-Aldrich) and all other chemicals (analytical grade): ammonium chloride (Analar®; product code: 100173D), calcium hydroxide (EMSURE® ACS, Reag. Ph Eur; CAS #: 1305-62-0), disodium edetate (Sigma-Aldrich® Laboratory Chemicals; lot number: 6381-92-6), and barium chloride (Suprapur®; CAS #: 10361-37-2).

Acetic acid (Sigma-Aldrich® Laboratory Chemicals; lot number: 72430), diphenylamine (LabChem®; product code: LC13610), and sulfuric acid (ReAgent®; batch number: 62042) were used.

#### 2.3.2. Procedures

HPLC analysis and UV-absorbance (UV-Visible spectrophotometer: Celil instruments CE 7200, Cambridge, England) at 210 and 254 nm and conductivity (*μ*S/cm) test (conductivity meter: HI9033; Hanna instruments, Portugal) and physicochemical tests [32] were conducted on different grade water (tap water, distilled water (before and/or after cleaning distillation unit), and fresh ultrapure water (18.2 MΩ × cm at 25°C)) produced in JuLaDQ. In addition, the consistent capability of the established water purification system of JuLaDQ was evaluated through routine monitoring of the critical quality parameters (i.e., physicochemical (Ph. Int.) tests, HPLC-DAD global peak area, and resistivity) of freshly prepared lab water for a period of one year (08/2018–07/2019). Moreover, pH of freshly prepared lab water was measured using a calibrated pH meter (Adwa-AD1020, UK). The pH was measured after adding 0.3 ml of saturated KCl into 100 ml of fresh lab water.

The physicochemical tests of lab water (limit tests for heavy metals, ammonia, calcium and magnesium, carbon dioxide, chloride, nitrate, sulfates, oxidizable matter, oxidizable matter, nonvolatile residue, and acidity or alkalinity) were conducted following the International Pharmacopoeia methods [32].

Resistivity of lab water was monitored online from Nanopure Analytical Ultrapure Water System (model number: D11901 (7143); Thermo Fisher Scientific).

The HPLC analysis of lab water (18.2 MΩ × cm at 25°C) was conducted using Agilent 1260 Infinity series HPLC system coupled with a C18 column (Waters Spherisorb^®^; ODS1: 4.0 mm × 250 mm, 5 *μ*m with guard column) and diode array detector (DAD). The mobile phase used was gradient elution (0–100%) of water (ultrapure)/acetonitrile (HPLC grade, Sigma-Aldrich) (Table 1). The flow rate and run time were 2.0 ml/min and 30 min, respectively. The HPLC analysis was performed at detection wavelengths of 210 and 254 nm [33]. In addition, HPLC analysis (at 254 nm) of lab water (18.2 MΩ × cm at 25°C) stored for three days at room temperature (15–30°C) in a soda lime glass (type III) (Wheaton, USA) container tightly covered with plastic stoppers was conducted. The cost for production of distilled and lab water (18.2 MΩ × cm at 25°C) per liter was estimated.

### 2.4. Data Analysis

The six-sigma limit of the data obtained from the routine quality analysis of resistivity, total chromatographic peak area, and pH of freshly prepared lab water was calculated using Microsoft Excel 2010.

## 3. Results

The predefined purpose of the pharmaceutical QC lab establishment was production of reliable analytical QC results, which are essential to take correct decisions on medicines. Therefore, those factors which affect the quality of analytical results were considered in the design and establishment of JuLaDQ. Since JuLaDQ is established with the main objective to contribute to the quality of medicines in the Horn of Africa region by QC-analytical activities (e.g., surveys, inspection-supporting, and industrial/governmental release of medicines), it has implemented a quality management system, based on World Health Organization (WHO), European Medicine Agency (EMA), and ISO/IEC17025: (2017) standards. WHO quality requirements implemented in JuLaDQ are presented in Table 2.

**Figure 1:**
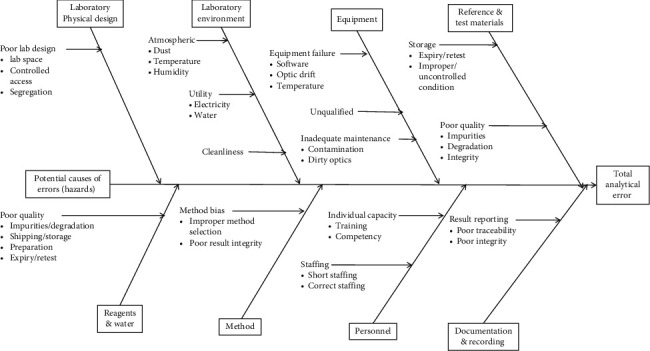
Ishikawa diagram for risk assessment in JuLaDQ laboratory.

The establishment of JuLaDQ applied the risk-based QbD principles. The target lab performance is compliance to quality standards set by WHO quality requirements to obtain the prequalification status. Similarly, lab quality attributes were defined and were analogous to pQbD and aQbD; the term lQbD is hence introduced. One example of the lQbD activity is the risk assessment, visualized by the Ishikawa (fishbone) diagram, used in the establishment of JuLaDQ (Figure 1).

**Figure 2:**
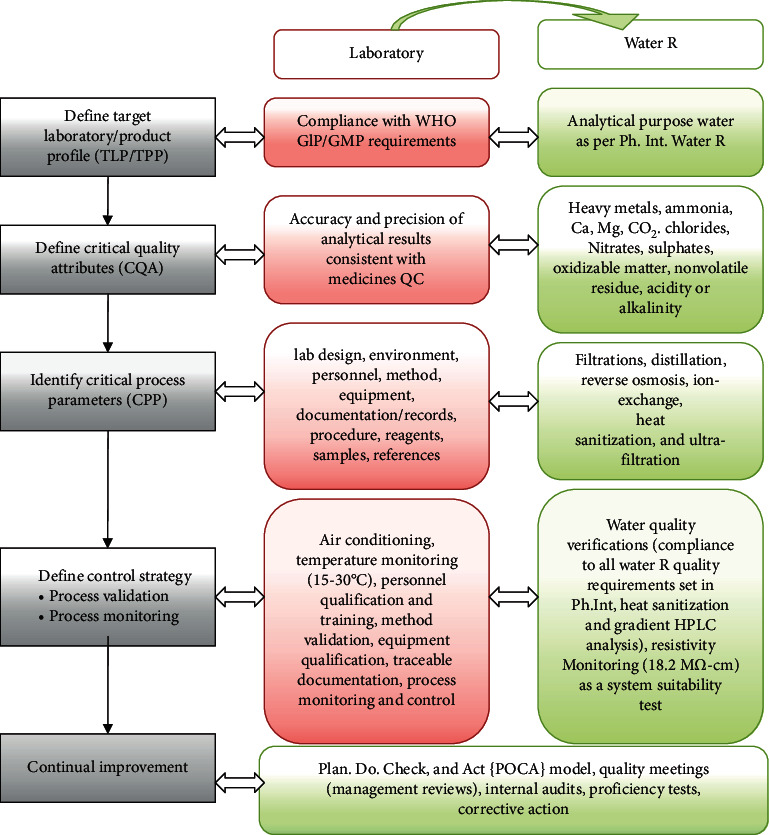
Lab QbD workflow and its application to lab water (GLP: good laboratory practice; GMP: good manufacturing practice).

The overall laboratory quality attributes (LQA) affecting quality of analytical results (lab performance) were found to be lab design, environment, sample, method, personnel, equipment, consumables, and quality control procedures. Accordingly, appropriate GLP/GMP is being maintained in JuLaDQ by implementing appropriate workflow of samples and test data according to the WHO standards (Table 2). Moreover, lab water was used as a typical but critical QbD-flow example (Figure 2) to demonstrate lQbD. Internationally recognized lab water quality standards define different types presented in Supplementary File 1.

**Figure 3:**
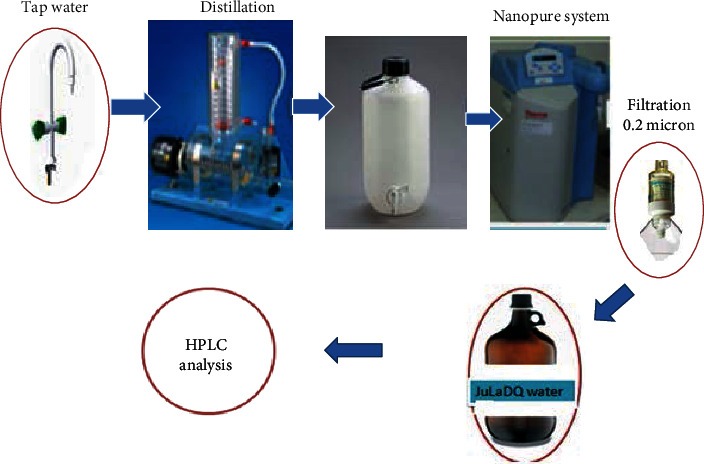
Customized JuLaDQ water purification system.

A customized water purification system combining different feasible and setting-suitable water purification system comprising distillation and Nanopure Analytical Ultrapure Water System combined with a 0.2-micron filter was set up (Figure 3.

The typical analytical quality test results of the three water types produced in JuLaDQ (tap water, distilled water, and purified water) according to Ph. Int. water *R* quality requirements are presented in Table 3. Overall analytical quality of different water grades produced in JuLaDQ is presented in Table 4. The results of the HPLC stability study for lab water (18.2 MΩ × cm at 25°C) is presented in Table 5.

The results of resistivity, HPLC global peak area (at 210 and 254 nm), and pH of fresh lab water (18.2 MΩ × cm at 25°C) produced in JuLaDQ are presented in Supplementary File 2.

**Figure 4:**
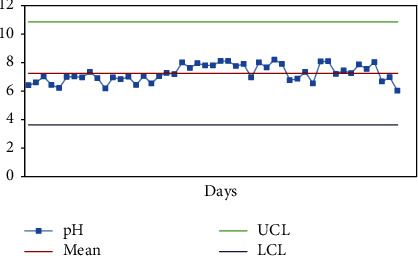
Control chart (six-sigma limits) indicating the trend of pH of lab water (18.2 MΩ × cm at 25°C) over time (*n* = 49 days/year). UCL: upper control limit; LCL: lower control limit.

**Figure 5:**
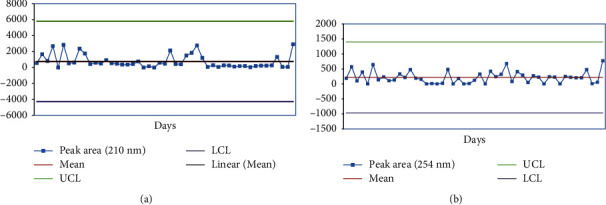
Control chart (six-sigma limits) indicating the trend of HPLC global peak area (a) at 210 nm and (b) 254 nm of fresh lab water (18.2 MΩ × cm at 25°C) over time (*n* = 49 days/year). UCL: upper control limit; LCL: lower control limit.

Price estimation for production of lab water is presented in Supplementary File 3. Control charts indicating the trends of HPLC global peak area at 210 and 254 nm and pH of fresh lab water (18.2 MΩ × cm at 25°C) are presented in Figures 4, 5(a) and 5(b), respectively.

## 4. Discussion

An important point in designing a pharmaceutical QC lab for improved quality based on the QbD principles is defining causes of variability and devising appropriate control strategies in order to reduce the associated risks of laboratory performance. The cornerstone concepts in such lQbD principles are target laboratory profile (TLP), laboratory quality attributes (LQA), risk assessment, critical process parameters (CPPs), control strategy, and continuous improvement.

Quality target profile (QTP) forms the basis of QbD, which is in relation to the predefined objective criteria. The concepts of analytical target profile (ATP) and target product profile (TPP) are described and defined in ICH Q8 [2] parallel lQbD's target laboratory profile (TLP). TLP is therefore the prospective summary of the quality characteristics of a QC laboratory that ideally will be achieved to ensure the desired quality standard. For a QC lab, it implies developing quality system based on regulatory requirement guidelines: good laboratory practices (GLP) and/or good manufacturing practices (GMP). TLP is the compliance to the requirements of good practices for pharmaceutical quality control laboratories (GPQCLs) set by WHO [34] supported mainly by the international standards ISO/IEC 17025 : 2017 [28], which is the prime target for JuLaDQ.

High quality material and consumables are critical for efficient and precise lab performance. In QC labs, water is used to prepare buffers, blanks, controls, sample solutions, and mobile phases in analytical procedures [35]. Thus, ensuring highest purity of water could help to reduce HPLC performance problems attributable directly to the quality of water used in preparing HPLC eluents, standards, and samples [36]. Therefore, using the appropriate water quality is of utmost importance in a resource-limited environment where the costs and handling also play a role. As our main objective is setting up a pharmaceutical QC lab according to WHO accepted standards, analytical water as defined in the Ph. Int. (water R) [32] is our minimum quality target.

For accurate and reliable analytical results obtained from pharmaceutical QC laboratories, water *R* according to Ph. Int. is critical since Ph. Int. methods are/will be mostly used in JuLaDQ. The general quality attributes for laboratory water were listed down to be conductivity/resistivity, turbidity, microbial content, endotoxins, and total organic carbon (TOC) [37–39]. However, TPP reveals that the product water should comply with water *R* requirements set in Ph. Int., and the LTP indicates that JuLaDQ is not meant to perform biological and/or microbiological tests; the CQAs for lab water are those attributes which are described in Ph. Int. Therefore, CQAs for lab water include heavy metals, ammonia, calcium and magnesium, carbon dioxide, chlorides, nitrates, sulphates, oxidizable matter, nonvolatile residue, and acidity or alkalinity [32].

To clearly define and identify critical water purification process parameters (CPPs), a number of practical experiments were conducted. The literature specifications for different water types revealed that no single water purification unit operation process could provide the TPP, analytical quality water *R* of the Ph. Int. Therefore, a customized water purification system combining different CPPs including filtration, distillation, and nanopure water purification technology was designed and installed.

Since biological and/or microbiological testing of medicines is currently not performed in JuLaDQ, microbial content and endotoxins tests were not defined to be current critical quality attributes. The practical experimental results (Table 4; Supplementary File 2) indicated that not only ultrapure water but also distilled water comply with the water *R* analytical quality specification set in Ph. Int., making cost estimation for production of both water types very demanding in such a resource-limited setting. Therefore, the QTP was evaluated in terms of not only the water quality target but also operational cost.

Cost to produce both distilled and ultrapure water was estimated with the assumption that equipment depreciates after five years (about 250 weeks) with 20 l water consumption per week in the actual setting. Hence, both equipment and operational cost per liter of water produced for each water *R* types was calculated providing the total cost per liter for each product (see Supplementary File 3). It is obvious that the cost of production of ultrapure water (3.2 USD/l), which also includes the cost for distilled water, is about five times higher than that of distilled water (0.6 USD/l). However, the water *R* quality specification set in Ph. Int. is the minimum requirement, and distilled water of JuLaDQ does not meet the quality requirements of water set in Ph. Eur. since its resistivity (1.9 MΩ. cm) is by far less than the minimum resistivity requirement (≥18 MΩ. cm). Moreover, the cost of purchasing HPLC-grade packed purified water is 60 USD/l, which is unimaginable in such a resource-limited setting. Therefore, production of the ultrapure water (resistivity = 18.2 MΩ cm), which complies with the resistivity requirement set in Ph. Eur., is very demanding, especially in the case of gradient HPLC systems.

In Ph. Int., most often only isocratic system is used for cost and ruggedness reasons. However, some analytical methodologies require gradient HPLC at longer retention times making the gradient system more demanding even in resource-limited settings. Moreover, since in the future, we need to include endotoxin test, the ultrapure equipment is able to produce water for bacterial endotoxin test (BET) according to Ph. Int. [32]. Therefore, even though there was considerable variation in the resistivity values (MΩ. cm) between the two water types, and since resistivity is not a formal quality specification for water *R* in Ph. Int., it is possible to conclude that the ultrapure water (resistivity = 18.2 MΩ. cm) could be preserved for gradient HPLC experiments and the proposed future BET, while the distilled water can be utilized for isocratic HPLC analysis, glassware cleaning, and rinsing analytical activities.

Standards and norms such as ASTM D1193 [40] specify that water be drawn and used within 8 h, which might not be practical in actual settings. Therefore, optimal time of use should be established and the pilot stability study results reveal that water *R* can be used for 48 h without degrading in its quality if stored under normal conditions upwards in a soda lime glass (type III) container tightly covered with plastic stoppers. This suggests that optimal time of use for water *R* could be 48 h unlike 8 h specified by ASTM.

In pQbD, control strategy is a planned set of controls derived from current product and process understanding that assures process performance and product quality. The controls can include parameters and attributes related to the product and inputs, facility and equipment operation conditions, in-process controls, finished product specifications, and the associated methods and frequency of monitoring and control [15, 41]. In aQbD, control strategy includes the system suitability tests (SSTs) and revalidation aspects whenever need. Similarly, appropriate SSTs are employed as control element to ensure that consistent quality of water *R* according to Ph. Int. is maintained. Routine SSTs for water *R* are currently not included in Ph. Int. but essential to ensure consistent product quality. Moreover, all the quality parameters indicated in the Ph. Int. are qualitative color reactions, and no quantitative specification limits are set. Therefore, proposing SSTs used in the routine control strategy for water *R* according to Ph. Int. is critical.

The experimental results with regard to the overall analytical quality evaluation of different water grades in JuLaDQ indicated that HPLC chromatograms (and the global peak area at wavelengths of 210 and 254 nm) and conductivity/resistivity are parameters that have strong discriminatory effect among different water types than UV-absorbance. In the chromatograms, it is observed that there was a rise in baseline, number, and size of peaks obtained across ultrapure, distilled (after and before cleaning), and tap water. Quality parameters like the global peak area (mAU∗s) at 210 and 254 nm, conductivity (*μ*S/cm) (reverse for resistivity (MΩ. cm)), and UV-absorbance (AU) are increasing in similar fashion, but with different rates. For example, the ratio of HPLC chromatogram global peak area (mAU∗s) at 254 nm for distilled water to ultrapure water was found to be 60.2, while the same ratio from the UV-absorbance (AU) was only 1.8 (Table 4). Similarly, the ratio of conductivity (*μ*S/cm) between tap and distilled water indicated a very significant figure (967.3). Similarly, ratio of resistivity between distilled and tap was 950. Therefore, both HPLC chromatogram global peak area (mAU∗s) (at 210 and 254 nm) and resistivity (MΩ. cm) at 25°C should be utilized as the routine SST parameters and need to be controlled.

Using the HPLC chromatograms global peak area (mAU∗s) at 210 and 254 nm, it is possible to effectively discriminate between different water qualities based on compliance to water *R* quality requirements set in Ph. Int. For example, the global peak area (mAU∗s) at 254 nm for distilled water that complies with water *R* (Ph. Int.) was 3,551, while that of tap water that failed the water *R* quality requirement was 8,520. Therefore, it is very logical to propose a specification limit of 5,000 mAU∗s for global peak area at 254 nm as an SST parameter. Similarly, we proposed a specification limit of 5,500 mAU∗s for the global peak area at 210 nm. These HPLC-UV SSTs could serve as an alternative for TOC (total organic carbon), which requires additional and expensive equipment.

According to ASTM, water type 2 is produced by distillation and is similar to distilled water in JuLaDQ, which complies with water *R* in Ph. Int. The resistivity (MΩ. cm) for water type 2 (= distilled water) is ≥ 1.0, a value which can be taken as a routine SST specification for water *R*.

The proposed specification limits (HPLC-UV global peak area< 5000 mAU∗s at 254 nm, 5500 mAU∗s at 210 nm; resistivity ≥1.0 MΩ. Cm) were followed as the control strategy for routine analysis of water quality produced in JuLaDQ. The results of routine analysis of Ph. Int. tests, HPLC analysis (at 210 and 254 nm), resistivity, and pH of fresh lab water suggest that water purification system of JuLaDQ is capable to consistently provide ultrapure water (ASTM, 1983). Six sigma control chart constructed based on the results of routine monitoring of HPLC global peak area at 210 and 254 nm of freshly prepared lab water (18.2 MΩ × cm at 25°C) revealed that the variations observed over time were within six-sigma control limit (Figure 5). The observed HPLC global peak area could be attributed to the presence of organic contaminants with increasing sensitivity at the UV detection wavelength of 210 and 254 [36].

Since freshly prepared high purity lab water have an advantage over commercially available HPLC grade water having relatively inferior results when used as eluent [41, 42], the established water purification system of JuLaDQ is critical in producing the desired quality of water. In addition, the cost estimation of the present study indicate that the established water purification system is important to produce highest purity lab water with relatively minimum operation cost (3.2 USD/l) compared with commercially available HPLC grade bottled water (60 USD/l).

Though resistivity and/or conductivity and TOC are the most commonly used quantitative specifications for the purity of lab water [40], utilizing HPLC global peak area at 210 and 254 nm, which serves to compare level of TOC in lab water [36] as the routine system suitability test (SST) parameter, is more productive in evaluating quality of lab water. The routine HPLC analysis of lab water revealed that the maximum HPLC global peak areas of 2911.9 and 772.7 mAU∗s were observed at 210 and 254 nm, respectively (Supplementary File 2). Thus, the proposed SSTs are logical and could be adapted as control limits. In addition, since organic contaminants could affect chromatographic assays and alter column performance in HPLC and LC-MS [38, 43], considering global peak areas as SST parameter is critical in maximizing chromatographic performance.

## 5. Conclusion

In the present study, the lQbD concept is introduced and applied in the establishment of lab water purification system in JuLaDQ. In addition, the results of routine HPLC analysis of lab water produced in JuLaDQ revealed that the proposed HPLC-UV specification limits could serve as discriminatory control strategy to evaluate the capability of water purification system and quality of water.

## Figures and Tables

**Table 1:** Gradient elution of the mobile phase.

*#*	Time (min)	% Water	% Acetonitrile
1	0.0	100.0	0.0
2	1.0	100.0	0.0
3	21.0	0.0	100.0
4	30.0	0.0	100.0

**Table 2:** WHO quality requirements implemented in JuLaDQ [34].

Quality attributes		Specifications
Personnel and organization	Personnel	Qualified, trained, and experienced
	Legal basis and organization	Legal establishment and proper organizational structure
Analytical workflow	Sampling	Appropriate sampling plan and sample documentation
	Samples	Samples unique identification and integrity during transport and storage
	Test results	Appropriate monitoring and evaluation
	Test reports	Include test results, and details of sample and test conditions
	Records	Data integrity and availability
	Methods	Proper validation
	Equipment	Calibration, servicing, and maintenance
	Lab environment	Temperature and humidity monitoring and control
	Documentation control	Written standard operating procedures for each activity
	Out-of-specifications	Corrective and preventive actions
	Customers	Complaint handling
	Contracts	Supplier and subcontractor management
	Quality audits	Continuous internal and external quality audits

**Table 3:** Typical analytical quality results of water *R* according to Ph. Int.

#	Test	Specification limit	Compliance (√)/noncompliance (x)		
			Ultrapure water	Distilled water^*∗*^	Tap water
1	Heavy metals	Color not darker than the same untreated purified water	√	√	x
2	Ammonia	Color of test solution is not more intense than standard solution	√	√	√
3	Calcium and magnesium	Pure blue color	√	√	√
4	Chlorides	Clear and colorless	√	√	√
5	Nitrates	No blue color appeared at the interface of the two liquids	√	√	√
6	Sulfates	Clear and colorless	√	√	√
7	Oxidizable matter	Faintly pink test solution	√	√	√
8	Nonvolatile residue	<0.001%	√	√	X
9	Alkalinity/acidity	No red color up on addition of methyl blue and no blue color appears up on addition of bromothymol blue	√	√	√

^*∗*^After cleaning.

**Table 4:** Overall analytical quality of different water grades in JuLaDQ using different parameters.

#	Water type	Typical chromatogram	Total peak area (mAU^*∗*^s)		UV-absorbance (AU)		Conductivity (*μ*S/cm) (resistivity in MΩ.cm)	Water *R* ph. Int. Compliance
			210 nm	254 nm	210 nm	254 nm		
1	Tap water	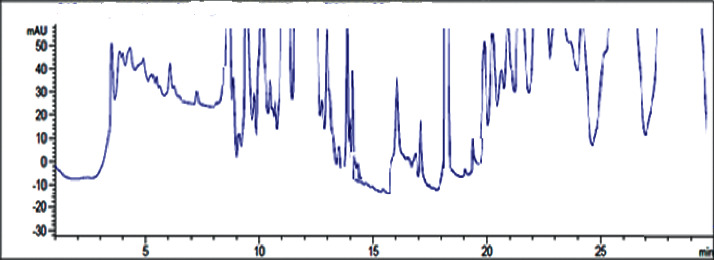	92435	8520	0.623	0.111	503 (0.002)	Did not comply
2	Distilled water before cleaning	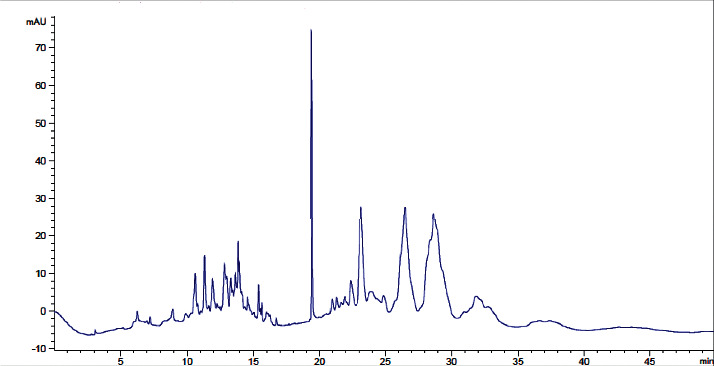	88206	7199	0.399	0.107	0.53 (1.9)	NA
3	Distilled water after cleaning	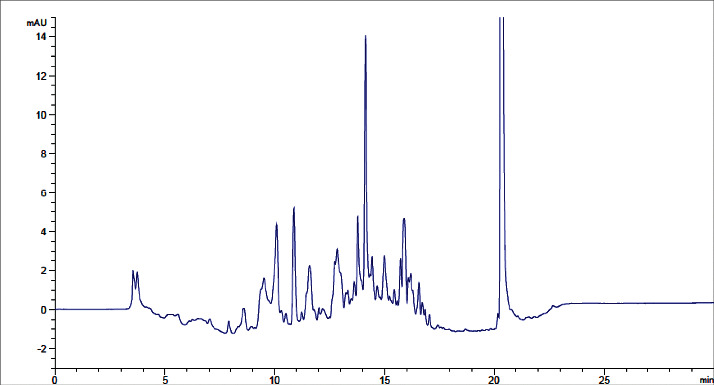	43384	3551	0.317	0.097	0.52 (1.9)	Complies
4	Ultrapure water	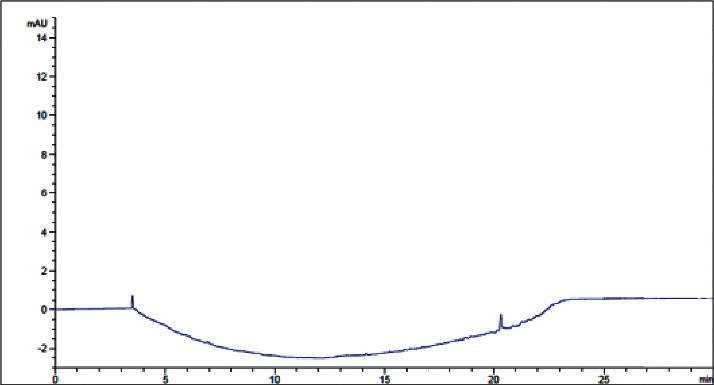	722	59	0.098	0.054	0.055 (18.2)	Complies

NA: not applicable.

**Table 5:** Pilot HPLC stability results of ultrapure water.

#	Experiment	Time (h)	Total peak area (mAU^*∗*^s) at 254 nm
1	Day 0	0	12.9
2	Day 1	24	12.8
3	Day 2	48	68.7

## Data Availability

The datasets generated during and/or analyzed during the current study are available from the corresponding author on reasonable request.

## Supplementary Materials

Supplementary Materials Supplementary File 1: general laboratory water types and specifications. Supplementary File 2: the results of pH, resistivity, and HPLC analysis of fresh lab water. Supplementary File 3: estimated cost per liter for production of water *R*.

## References

[1] Izat N., Yerlikaya F., Capan Y. (2014). A glance on the history of pharmaceutical quality by design. *OA Drug Design and Delivery*.

[2] International Conference on Harmonization ICH Q8(R2) (2009). *International Conference on Harmonisation Of Technical Requirements for Registration of Pharmaceuticals for Human Use*.

[3] FDA (2014). *Pharmaceutical Quality Control Labs (7/93), Guide to Inspections of Pharmaceutical Quality Control Laboratories*.

[4] Lionberger R. A., Lee S. L., Lee L., Raw A., Yu L. X. (2008). Quality by design: concepts for ANDAs. *The AAPS Journal*.

[5] Yu L. X. (2008). Pharmaceutical quality by design: product and process development, understanding, and control. *Pharmaceutical Research*.

[6] Orlandini S., Pinzauti S., Furlanetto S. (2013). Application of quality by design to the development of analytical separation methods. *Analytical and Bioanalytical Chemistry*.

[7] Monks K., Molnár I., Rieger H.-J., Bogáti B., Szabó E. (2012). Quality by Design: multidimensional exploration of the design space in high performance liquid chromatography method development for better robustness before validation. *Journal of Chromatography A*.

[8] Vogt F. G., Kord A. S. (2011). Development of quality-by-design analytical methods. *Journal of Pharmaceutical Sciences*.

[9] Borman P., Nethercote P., Chatfield M., Thompson D., Truman K. (2007). The application of quality by design to analytical methods. *Pharmaceutical Technology*.

[10] Peraman R., Bhadraya K., Reddy Y. P. (2015). Analytical quality by design: a tool for regulatory flexibility and robust analytics. *International Journal of Analytical Chemistry*.

[11] Raman N. V. V. S. S., Mallu U. R., Bapatu H. R. (2015). Analytical quality by design approach to test method development and validation in drug substance manufacturing. *Journal of Chemistry*.

[12] Reid G. L., Morgado J., Barnett K. (2013). Analytical quality by design (AQbD) in pharmaceutical development. *American Pharmaceutical Review*.

[13] Bracke N., Barhdadi S., Wynendaele E., Gevaert B., D’Hondt M., De Spiegeleer B. (2015). Surface acoustic wave biosensor as a functional quality method in pharmaceutics. *Sensors and Actuators B: Chemical*.

[14] (2006). *International Conference on Harmonization (ICH) Q9*.

[15] (2008). *International Conference on Harmonization (ICH) Q10*.

[16] Yao H., Vancoillie J., D’Hondt M., Wynendaele E., Bracke N., Spiegeleer B. D. (2016). An analytical quality by design (aQbD) approach for a l -asparaginase activity method. *Journal of Pharmaceutical and Biomedical Analysis*.

[17] Korakianiti E., Rekkas D. (2011). Statistical thinking and knowledge management for quality-driven design and manufacturing in pharmaceuticals. *Pharmaceutical Research*.

[18] Schweitzer M. G., Pohl M., Hanna-Brown M. (2010). Implications and opportunities of applying QbD principles to analytical measurements. *Pharmaceutical Technology*.

[19] International Conference on Harmonization (ICH) Q2(R1) (2005). *Validation of Analytical Procedures: Text and Methodology*.

[20] Collins W. (2013). *Managing Quality: The Role of Lab Managers’ Role in Building a Robust, Reliable Analytical Quality System*.

[21] Garfield F. M., Klesta E., Hirsch J. (2000). *Quality Assurance Principles for Analytical Laboratories*.

[22] Kuselman I., Pennecchi F., Fajgelj A., Karpov Y. (2013). Human errors and reliability of test results in analytical chemistry. *Accreditation and Quality Assurance*.

[23] Ellison S. L. R., Hardcastle W. A. (2012). Causes of error in analytical chemistry: results of a web-based survey of proficiency testing participants. *Accreditation and Quality Assurance*.

[24] Person N. B. (2013). Developing risk-based quality control plans. *Clinics in Laboratory Medicine*.

[25] Krouwer J. S., Cembrowski G. S. (2011). Towards more complete specifications for acceptable analytical performance – a plea for error grid analysis. *Clinical Chemistry and Laboratory Medicine*.

[26] Suleman S. (2016). Quality of selected medicines in Ethiopia: Analytical and Regulatory contribution, Chapter 2 Quality-by-design principles applied to the establishment of a pharmaceutical quality control laboratory in a resource-limited setting: the lab-water.

[27] World Health Organization (WHO) (2019). *Essential Medicines and Health Products: Prequalification of Medicines. Quality Control Laboratories*.

[28] International Organization for Standardization (2017). *General Requirements for the Competence of Testing and Calibration Laboratories, ISO/IEC 17025:2017*.

[29] United States Food and Drug Adminstration (2004). *Final Report on Pharmaceutical cGMPs for the 21st Century: A Risk-Based Approach*.

[30] Ishikawa K., Loftus J. H. (1990). *Introduction to Quality Control*.

[31] World Health Organization (2015). *WHO Prequalification Team, WHO Inspection Report*.

[32] (2018). *The International Pharmacopoeia - Eighth Edition. Reagents, test solutions and volumetric solutions: W – Water R*.

[33] Regnault C., Kano I., Darbouret D., Mabic S. (2004). Ultrapure water for liquid chromatography-mass spectrometry studies. *Journal of Chromatography A*.

[34] World Health Organization (2010). *WHO Good Practices for Pharmaceutical Quality Control Laboratories*.

[35] Nabulsi R., Al-Abbadi M. A. (2014). Review of the impact of water quality on reliable laboratory testing and correlation with purification techniques. *Laboratory Medicine*.

[36] Mabic S., Regnault C., Krol J. (2005). *The Misunderstood Laboratory Solvent: Reagent Grade Water for HPLC*.

[37] Taverniers I., De Loose M., Van Bockstaele E. (2004). Trends in quality in the analytical laboratory. I. Traceability and measurement uncertainty of analytical results. *TRAC Trends in Analytical Chemistry*.

[38] Stewart B. M. (2000). The production of high-purity water in the clinical laboratory. *Laboratory Medicine*.

[39] National Institute of Health (2013). *Laboratory Water: Its Importance and Application*.

[40] American Society of Testing and Materials (ASTM) (1983). *Standard Specifications for Reagent Water*.

[41] Jorgen B. (2013). Process understanding: for scale-up and manufacture of active ingredients.

[42] ELGA (2011). Application Note: Ultrapure water enables excellent chromatographic performance for LC-MS analysis. https://www.elgalabwater.com/sites/default/files/2018-10/elga-application-note-hplc-and-uhplc%20%281%29.pdf.

[43] ELGA (2011). Application note, Type I ultrapure water crucial for HPLC and UHPLC. http://www.iptonline.com/articles/public/2.pdf.

