# Supplementary material for: Quality-by-Design Principles Applied to the Establishment of a Pharmaceutical Quality Control Laboratory in a Resource-Limited Setting: The Lab Water
Source: Int J Anal Chem. 2022 Apr 21;2022:2062406. doi: 10.1155/2022/2062406 (PMC9050335; doi:10.1155/2022/2062406)
Supplement: Supplementary Materials — Supplementary File 1: general laboratory water types and specifications. Supplementary File 2: the results of pH, resistivity, and HPLC analysis of fresh lab water. Supplementary File 3: estimated cost per liter for production of water R. [file 2062406.f1.docx]

**Additional file 1**

**Table**. General laboratory water types and specifications according to different standards.

| ***Parameter (unit)*** | ***Standards and specifications*** | | | | | | | | | | | |
| --- | --- | --- | --- | --- | --- | --- | --- | --- | --- | --- | --- | --- |
|  | ***American Society for Testing and Materials (ASTM)*** | | | | ***National Committee for Clinical Laboratory Standards (NCCLS)*** | | | ***College of American Pathologists (CAP)*** | | | ***^e^Ph. Eur.*** | |
|  | ***Type 1*** | ***Type 2^a^*** | ***Type 3*** | ***Type 4*** | ***Type I*** | ***Type II*** | ***Type III*** | ***Type I*** | ***Type II*** | ***Type III*** | ***^c^Water R*** | ***Water for chromatography*** |
| Conductivity at 25 ˚C (μS/cm) | 0.056 | 1.0 | 0.25 | 5.0 | 0.1 | 1 | 10 | 0.1 | 0.5 | 10.0 | 5.1 | Deionized water R with a resistivity not less than 18 MΩ.cm |
| Resistivity at 25 ˚C (MΩ.cm) | 18.0 | 1.0 | 4.0 | 0.2 | 10 | 1 | 0.1 | 10.0 | 2.0 | 0.1 | 0.2 |  |
| TOC (μg/l) | 100 | 50 | 200 | NL | 50 | 200 | 1000 | NS | NS | NS | 500 |  |
| Sodium (µg/l) | 1 | 5 | 10 | 50 | NS | NS | NS | NS | NS | NS | NS |  |
| Chloride (µg/l) | 1 | 5 | 10 | 50 | NS | NS | NS | NS | NS | NS | ^d^Colour reaction |  |
| Silica (µg/l) | 3 | 3 | 500 | NL | 5 | 10 | 100 | 5 | 100 | 1000 | NS |  |
| Total solids (mg/l) | NS | NS | NS | NS | 0.1 | 1 | 5 | NS | NS | NS | NS |  |
| Bacteria (CFU/ml) | _b_ | | | | 10 | 1000 | NS | 10 | 1000 | NS | 100 |  |
| Endotoxins (IU/ml) |  |  |  |  | NS | NS | NS | NS | NS | NS | 0.25 |  |

TOC: total organic carbon, NL: no limit, NS: not specified

^a^Prepared by distillation (= Distilled water). ^b^When bacteria levels need to be controlled, ASTM further classifies reagent grade water types into type A (bacteria: 1CFU/100 ml, endotoxins: 0.03 EU/ml), type B (bacteria: 10CFU/100 ml, endotoxins: 0.25 EU/ml), type C (bacteria: 1000 CFU/100). ^c^Water R: purified water in Ph. Eur. with additional specifications for heavy metals: 0.1 ppm, nitrates: 0.2 ppm, aluminium: 10 ppb, and ammonium: 0.2 ppm. ^d^The colour from solution prepared by addition of water R (10 ml) + dilute nitric acid R (1 ml) + silver nitrate solution R2 shows no change in appearance for at least 15 min.

^e^Other R-types described in the Ph. Eur. for special analytical purposes include: water, distilled (water R prepared by distillation); water, distilled, deionized (deionized water R prepared by distillation with a resistivity 18 MΩ.cm); water, ammonium-free; water, carbon dioxide-free (water R boiled for a few minutes and protected from the atmosphere during cooling and storage or deionized water R with resistivity 18 MΩ.cm); water, nitrate-free; and water, particle-free (water R filtered through a membrane with a pore size of 0.22 μm).

**Additional file 2**

**Table** . The results of pH, resistivity and HPLC analysis of fresh lab water in JuLaDQ.

| **#** | **Resistivity**  **(mΩ-cm)** | **pH** | **HPLC Analysis** | | |
| --- | --- | --- | --- | --- | --- |
|  |  |  | **254 nm** | **210 nm** | |
| 1 | 18.2 | 6.40 | 185.64 | | 561.70 |
| 2 | 18.2 | 6.60 | 571.48 | | 1654.59 |
| 3 | 18.2 | 7.02 | 100.16 | | 811.53 |
| 4 | 18.2 | 6.43 | 396.22 | | 2680.97 |
| 5 | 18.2 | 6.21 | 0.00 | | 0.00 |
| 6 | 18.2 | 6.99 | 643.24 | | 2839.44 |
| 7 | 18.2 | 7.02 | 135.10 | | 521.80 |
| 8 | 18.2 | 6.95 | 235.30 | | 621.90 |
| 9 | 18.2 | 7.34 | 106.57 | | 2361.87 |
| 10 | 18.2 | 6.90 | 130.89 | | 1756.74 |
| 11 | 18.2 | 6.18 | 332.27 | | 419.74 |
| 12 | 18.2 | 6.96 | 215.50 | | 601.70 |
| 13 | 18.2 | 6.82 | 479.35 | | 491.1 |
| 14 | 18.2 | 7.00 | 190.76 | | 918.41 |
| 15 | 18.2 | 6.43 | 155.40 | | 541.60 |
| 16 | 18.2 | 7.05 | 0.00 | | 457.55 |
| 17 | 18.2 | 6.54 | 9.67 | | 365.63 |
| 18 | 18.2 | 7.04 | 0.00 | | 357.68 |
| 19 | 18.2 | 7.27 | 17.33 | | 429.28 |
| 20 | 18.2 | 7.18 | 480.09 | | 758.00 |
| 21 | 18.2 | 8.00 | 0.00 | | 0.00 |
| 22 | 18.2 | 7.63 | 183.66 | | 145.05 |
| 23 | 18.2 | 7.95 | 0.00 | | 0.00 |
| 24 | 18.2 | 7.80 | 10.40 | | 605.08 |
| 25 | 18.2 | 7.80 | 122.15 | | 466.50 |
| 26 | 18.2 | 8.10 | 328.50 | | 2116.9 |
| 27 | 18.2 | 8.10 | 0.00 | | 425.00 |
| 28 | 18.2 | 7.75 | 421.00 | | 399.00 |
| 29 | 18.2 | 7.89 | 239.00 | | 1487.00 |
| 30 | 18.2 | 6.95 | 318.88 | | 1831.54 |
| 31 | 18.2 | 8.00 | 680.70 | | 2763.10 |
| 32 | 18.2 | 7.65 | 78.08 | | 1195.42 |
| 33 | 18.2 | 8.20 | 408.82 | | 63.30 |
| 34 | 18.2 | 7.90 | 293.71 | | 281.07 |
| 35 | 18.2 | 6.77 | 49.30 | | 58.50 |
| 36 | 18.2 | 6.86 | 270.00 | | 266.00 |
| 37 | 18.2 | 7.34 | 223.97 | | 239.05 |
| 38 | 18.2 | 6.54 | 0.00 | | 99.85 |
| 39 | 18.2 | 8.07 | 239.80 | | 165.14 |
| 40 | 18.2 | 8.08 | 227.26 | | 178.29 |
| 41 | 18.2 | 7.21 | 0.00 | | 30.50 |
| 42 | 18.2 | 7.43 | 250.80 | | 181.80 |
| 43 | 18.2 | 7.25 | 221.70 | | 228.80 |
| 44 | 18.2 | 7.86 | 206.20 | | 226.90 |
| 45 | 18.2 | 7.55 | 206.20 | | 266.90 |
| 46 | 18.2 | 8.02 | 476.26 | | 1322.03 |
| 47 | 18.2 | 6.67 | 6.80 | | 70.17 |
| 48 | 18.2 | 6.97 | 55.90 | | 56.50 |
| 49 | 18.2 | 6.02 | 772.74 | | 2911.9 |

0.00: No peak observed

# (over time, number of days)

**Additional file 3**

**Table.** Estimated cost per liter for production of water R (distilled and ultrapure water) in JuLaDQ.

| ^1^**Water R (Ph. Int.)** | **Equipment name** | **Equipment cost (USD)** | ^1^**Equipment cost/l (USD)** | **Operation cost (USD)** | | | **Subtotal cost/l (USD)** | ^2^**Total cost/l (USD)** |
| --- | --- | --- | --- | --- | --- | --- | --- | --- |
|  |  |  |  | **Electricity cost/l** | **Consum-ables/l** | **Operator/l** |  |  |
| Distilled water | Merit Still, W4000 (Bibby Scientific, UK) | 1,270.00 | 0.3 | 0.013 | 0.0001 | 0.3 | 0.6 | 0.6 |
|  |  |  |  |  |  |  |  |  |
|  |  |  |  |  |  |  |  |  |
| Ultra Pure water | Nanopure Analytical ultrapure water system, Model # 7143 (Thermo Scientific, USA) | 8,500.00 | 1.7 | 0.0001 | 0.4 | 0.5 | 2.6 | 3.2 |
|  |  |  |  |  |  |  |  |  |
|  |  |  |  |  |  |  |  |  |
| HPLC grade packed water |  | | | | | | | ^3^60 |

^1^Five years (250 weeks) depreciation and 20 l water/week was used for calculation. ^2^The total cost to produce 1l ultrapure water includes cost of distilled water/l since distilled water is used as feed water. ^3^HPLC-gradient grade packed water/l costs 60 USD.
